# Remote feedback in endovascular simulation training: a mixed-methods study

**DOI:** 10.1186/s41077-024-00297-0

**Published:** 2024-06-11

**Authors:** Adam F. Roche, Daragh Moneley, Tim Lawler, Emily Boyle, Greg Gosi, Adrian O’Callaghan, Caitriona Cahir, Dara O’Keeffe, Claire M. Condron

**Affiliations:** 1 RCSI University of Medicine and Health Sciences, Dublin, Ireland; 2 Beaumont Hospital, Dublin, Ireland; 3 Tallaght University Hospital, Dublin, Ireland; 4 University Hospital Waterford, Waterford, Ireland; 5 James’s Hospital, Dublin, Ireland; 6 RCSI SIM Centre for Simulation Education & Research, 26 York Street, Dublin 2, D02 P796 Ireland

**Keywords:** Endovascular, Virtual reality, Simulation, Education, Patient safety

## Abstract

**Background:**

There is an increasing need to increase simulation-based learning opportunities for vascular surgery residents in endovascular skills training. This study aims to explore the effectiveness of remote expert instructional feedback of endovascular simulation-based education, as a means of increasing training opportunities in this area for vascular surgery residents.

**Methods:**

A mixed-methods study design was adopted. Twelve vascular surgery residents from Ireland were tasked with completing two endovascular renal artery procedures: one with in-person expert feedback and the other with remote instruction. Participants ranged in experience levels from second year to final year of residency. Following the training activities, interviews and a questionnaire were employed to gather information on the usefulness of remote feedback.

**Results:**

There was no significant difference reported by participants using a post-event validated questionnaire between remote and in-person feedback. During the interviews, participants expressed mixed feelings about the presence of the educator while practicing, but they eventually saw no limiting factors to their practice when the trainer provided remote feedback. When receiving performance feedback remotely, clear communication and a shared knowledge of the task development are critical to success.

**Conclusions:**

We believe these findings can inform the design and development of remote learning and assessment of endovascular skills training and ultimately provide increased opportunities for more skills practice for vascular surgical residents.

**Supplementary Information:**

The online version contains supplementary material available at 10.1186/s41077-024-00297-0.

## Background

Vascular surgical training has historically followed the Halstedian model of “see one, do one, and teach one” [1]. However, many contend that this approach to training is no longer appropriate [2, 3]. Advances in medical simulation technology and a greater recognition of the performance-based component of clinical competency, a focus on patient safety, and the need for efficient patient turnover, which restricts training time and reduces exposure to less common procedures, have all contributed to the increasing focus on simulation techniques [4]. Simulation-based education (SBE) complements endovascular (EV) technique practice by offering a safe environment for skill improvement through repetition. Vascular surgery residents attain competencies in a simulated setting with dedicated supervision and feedback [5]. Training using virtual reality (VR) simulators improves performance of both novice and experienced surgeons [6–8].

The provision of SBE for vascular surgical training programmes is hindered by insufficient resources [9]. Representing the intricacies of complex EV procedural tasks through a simulation modality other than VR is challenging [10], and VR platforms typically incur extensive programming, development, and subsequent high cost. These resources are then typically pooled centrally, frequently accommodated in off-site specialised simulation training centres, requiring both residents and trainers to travel in order to engage training. Barriers to establishing EV SBE programmes are mainly the cost of equipment, scheduling busy residents for off-the-job training and the availability of expert instructors. Traditional in-person observation and proctoring are a resource-intensive undertaking for surgical faculty. There is a need to think creatively and devise alternative solutions to maximise trainer involvement [11]. A more fluid training paradigm, which allows expert trainers to deliver feedback remotely on a more ad hoc basis inside and outside of core business hours, will increase residents training opportunities without missing vital performance feedback. Remote proctoring mitigates issues faced with geographical location and opens up a larger pool of national and international expert trainers as educators on SBE programmes [12]. Flexibility and scalability are advantages that technology can provide for remote coaching. However, in remote learning situations, the personal connections, gestures, and mentoring style that come with working in person may be impeded [13].

There has been debate about whether expert feedback is necessary when high-fidelity simulators provide accurate output metrics on learner performance. However, studies found that participants who received expert feedback during simulated EV tasks made fewer technical errors than those who did not receive expert feedback [4]. Effective evaluation of performance should combine simulator-derived metrics with live objective feedback, and not rely solely on the former [8].

During the coronavirus epidemic, social distance constraints provided chances to study the feasibility and usability of various learning platforms [14, 15]. As a pilot, we aimed to explore the educational effectiveness of remote instructional support and feedback of EV SBE. Based on these findings, inferences for the rollout of such remote-based learning programmes will be discussed.

## Methods

**Fig. 1:**
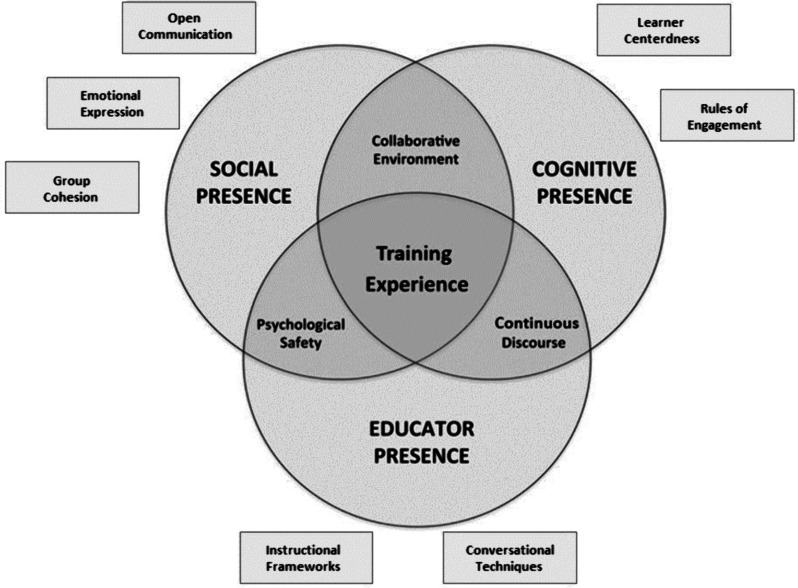
The modified CoI framework utilised to develop psychomotor skills in remote EV simulation training

A mixed-methods study design [16] was employed using semi-structured interviews and a post-task questionnaire. Additionally, as best practices, we used the standard guidelines in the design and implementation of this interventional research study [17, 18]. A modified version of the community of inquiry (CoI) framework [19] (Fig. 1) was used as a model to aid remote feedback and was discussed with faculty in meetings prior to the initial data collection session. The CoI framework demonstrates how social, cognitive, and educator presence interact to shape the overall remote learning experience. In summary, social presence considers personal attributes, whereas cognitive presence considers how educator and learners collaborate to identify problems and exchange ideas. Finally, educator presence examines how educators construct learning activities and engage in meaningful discourse. This framework is especially relevant in our study because the attributes of a successful surgical trainer [20] correlate well with the three domains of the CoI framework. We utilised a modified CoI questionnaire for the quantitative analysis segment [21].

### Materials

**Fig. 2:**
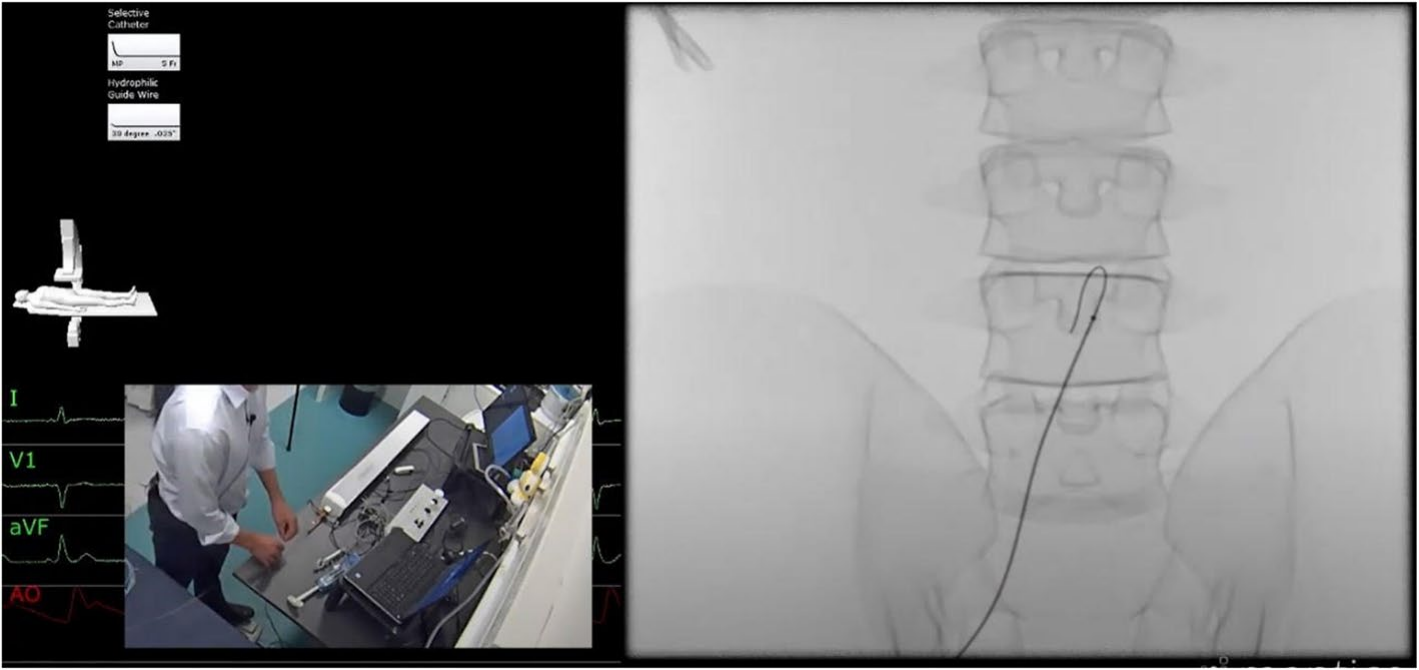
Interface display of the simulated task and environment, highlighting what faculty can see when remote

The simulator used in this study was a Mentice™ VIST G5 EV simulator, and the audio-visual (AV) software used to capture one-way live visual footage of the procedural tasks was CAE™ LearningSpace (Fig. 2). The telephones used as communication mediums between participants and trainers were Mitel 6867i SIP desktop phones. All data collection sessions took place in RCSI Simulation (SIM) Centre for Simulation Education and Research.

### Facilitators

#### Expert clinical facilitators

Experienced educators and consultant vascular surgeons (DM, EB, GG, AO’C) (*n* = *4*) volunteered to give participant feedback in this study, all of whom are working in Ireland and actively perform EV procedures as part of their practice. In order to integrate feedback practices, establish and preserve a psychologically safe learning environment, and apply the CoI framework to this study, the four facilitators and the remaining research team convened a number of group meetings prior to data collection.

#### Non-clinical facilitators

An experienced senior simulation technician who has extensive knowledge of the equipment (TL) assisted participants throughout the procedural tasks.

### Participants

Participants were non-consultant hospital doctors (NCHD) working in the field of vascular surgery, enrolled in the vascular surgery training programme at RCSI (*n* = *12*). The participants’ experience ranged from second year to seventh year of residency. This surgical specialty has a limited resident population in the Republic of Ireland; consequently, participants were recruited via e-mail using a convenience sample technique. This sample size is deemed a reasonable figure where the target population is homogenous [22]. Demographical participant data is shown in Table 1.

**Table 1:** Participant demographics

Number	12
Sex (F:M)	6:6
Age median (range)	30
Total *RRAS observed/assisted	8
Total *RRAS performed as primary operator	2

^*^Right renal artery stenosis

### Intervention design

Participants were divided into two independent groups, with their degrees of experience dispersed evenly across both groups. Group 1 performed the required tasks with the trainer on-site giving feedback in-person first. The training was then repeated with remote instructional support and feedback. Group 2 performed the tasks with remote instructional support and feedback first, followed by in-person trainer support at the second event. There was a short 5-min recess between sessions to allow the facilitator to relocate. This crossover design was used to promote a more in-depth discussion in subsequent interviews on perceptions of mutual discourse between trainer and participant, as well as how nuanced social characteristics were portrayed remotely and how these influenced learning. To allow time for familiarisation prior to task commencement, all participants were informed of the procedure they would be required to perform.

During the in-person instructional support and feedback sessions, the trainer remained in close proximity to the participants within the room where the simulation took place. Trainers relocated to a room on a different level in the simulation centre to provide feedback remotely via telephone, in the remote session.

### Simulated training procedures

The case that was undertaken by each participant on both occasions was a right renal artery stenosis (proximal). This procedure was chosen as it is relatively uncommon [23], thus providing a learning opportunity for participants to treat pathologies that they are less likely to encounter as a training opportunity in the operating room (OR). In this simulation, the patient case had normal renal anatomy with non-ostial disease. Access was already established within the simulator at the right femoral artery. The case included a preoperative (pre-op) angiogram of the right renal artery, providing a clear image of the anatomy and the location of the lesion. One simulation technician (TL) assisted the participants in the procedures, preparing and manoeuvring tools, moving the C-arm and overlaying roadmaps, and also dealing with any technical challenges that were encountered within the simulator. A task-specific checklist was developed (Appendix A) to guide informal oral feedback phase after task completion. This checklist was a modified version of the output metrics from the simulator [24], generating a total of 21 procedural steps, with a score of 0 indicating suboptimal, 1 indicating adequate, and 2 indicating good. This measure also included an overall competency score, which ranged from 0 (not competent) to 1 (borderline) to 2 (pass). All four expert facilitators agreed to the criteria set out, thus supporting internal consistency.

### Interviews

After the event, one author (A. R.) conducted the face-to-face semi-structured interviews, which took place immediately after the participants completed the second attempt of the procedural task. The interviews were audio-recorded and transcribed verbatim. The interview schedule was developed using the CoI framework. The interview schedule was tested with another author (T. L.) prior to data collection.

### Interview analysis

The framework method [25] in data analysis was used to analyse data elicited from the semi-structured interviews. A deductive approach was taken, informed by a modified CoI framework. This framework was modified somewhat inductively to reflect the content of the data set, including the revision of some of the existing themes (Fig. 1). For example, one author (A. R.) extracted salient discussion points from data sets and grouped them into the relevant theme from each of the three elements of the CoI framework. Each discussion point was subsequently grouped into an existing or revised subtheme/component from the CoI framework. Two further authors (C. C., T. L.) collaboratively coded all discussion points, and an agreed-upon coding structure was resolved through discussion. Theoretical sufficiency was evident after all interviews had ceased. Interview data was represented using core narratives, as per recommendations in the literature [26].

### Questionnaire

After all training events for both groups had concluded, participants and facilitators were sent a modified CoI framework survey instrument hosted on Google Forms, which they all completed within 7 days. The questions asked in this survey related to the delivery of the remote feedback sessions. Five items were included for each of the three elements (educator presence, social presence, and cognitive presence) (Table 2). Participants completed all three elements; however, facilitators only completed the social and cognitive presence sections. Items from the original survey instrument were either combined or removed if they were considered not relevant to this study. Items such as the involvement of other course participants, for example, were deemed irrelevant. Responses were scored using an ordinal scale (1 = strongly disagree) to (5 = strongly agree).

**Table 2:** Median (IQR) responses for the selected educator, social and cognitive presence items from the CoI survey and by the different learning groups and facilitators

Community of Inquiry Survey Instrument	Median (IQR) Overall	Median (IQR) Participant group 1 (*N* = 6)	Median (IQR) Participant group 2 (*N* = 6)	Median (IQR) Facilitators (*N* = 4)
**Educator presence**				
1. Trainer clearly communicated procedural steps	4 (4, 5)	4.5 (4, 5)	4 (4, 5)	
2. Trainer was helpful in guiding me towards understanding the simulated renal intervention that helped me clarify my thinking	4 (4, 4.5)	4 (4, 4)	4 (4, 5)	
3. Trainer helped to keep me engaged and participating in productive dialogue	4.5 (4, 5)	4.5 (4, 5)	4.5 (4, 5)	
4. Trainers actions reinforced the development of a sense of community amongst us	4.5 (3, 5)	4 (3, 5)	5 (3, 5)	
5. Trainer provided feedback that helped me understand my strengths and weaknesses relative to the task at hand	4.5 (4, 5)	4.5 (4, 5)	4.5 (4, 5)	
**Social presence**				
6. Online or web-based communication is an excellent medium for social interaction in learning simulated endovascular skills	4 (2, 5)	4 (2, 5)	4 (3, 4)	3.5 (3, 5)
7. I felt comfortable conversing through the online medium	4 (2, 5)	4.5 (4, 5)	4 (3, 5)	4 (3, 5)
8. I felt comfortable interacting with the trainer/trainee	4.5 (2, 5)	5 (4, 5)	4 (3, 5)	4 (3, 5)
9. I felt that my point of view was acknowledged by the trainer/trainee	4 (2, 5)	5 (4, 5)	4.5 (4, 5)	4 (3, 5)
10. Virtual learning of endovascular techniques through simulation training helps me to develop a sense of collaboration	4 (2, 5)	5 (5, 5)	4 (3, 4)	4 (3, 5)
**Cognitive presence**				
11. I felt motivated to explore questions related to the procedural task	4 (3, 5)	4 (4, 5)	4 (4, 4)	3.5 (3, 4)
12. Virtual discussion was valuable in helping me appreciate different perspective	4 (3, 5)	4 (4, 5)	4 (4, 4)	3.5 (3, 4)
13. Combining new information aided in task completion	4 (3, 5)	4 (4, 5)	4 (3, 5)	4 (3, 4)
14. Virtual learning activities helped me construct explanations/solutions	4 (3, 5)	4.5 (4, 5)	4 (4, 4)	4 (3, 4)
15. I aided in developing solutions throughout the task that can be applied in practice	4 (3, 5)	4 (4, 5)	4 (4, 5)	3.5 (3, 4)

### Questionnaire analysis

The overall median and interquartile range (IQR) was calculated for each of the 15 items within the survey instrument and for each of the three elements (educator, social and cognitive presence). The median (IQR) for the facilitator group, and participant groups 1 and 2, was also calculated separately for the three elements (educator, social and cognitive presence). The data was analysed using Stata Version 17.0 (StataCorp, College Station, TX, USA).

## Results

### Interviews

Twelve interviews were conducted with vascular surgery residents based in the Republic of Ireland. Interviews ranged in length from 16 to 61 min. We identified distinctive characteristics of remote feedback in EV skills training based on our inductive and deductive examination of those categories.

#### Interview: formulating discourse

This component emphasises the key role of the educator in structuring discussion for learning and building understanding through reflective discourse.

Participants had mixed feelings of apprehension and ambivalence upon commencement of their tasks in the remote courses, amplified when the trainer was not present. The remote environment, in particular, made some participants feel “more nervous” (P2), under the expectation that it would be “more difficult” (P3) than first envisaged. Other participants felt more calm with physical isolation from the trainer, “having more time to think when you’re a bit further removed” (P8).

Participants describe the use of impactful dialogue between trainer and participant to promote self-sufficiency and to identify solutions to achieve task completion during the remote course. One participant remarked that faculty were “prompting me to try and think and navigate better” (P7). Where applicable, faculty advocated an individualist approach to problem-solving throughout the procedural tasks, with feelings of independent troubleshooting and efficacious task progression likely to increase learner confidence. Furthermore, when participants required a more candid coaching style, mutual discourse produced decisive results to enhance task progression and completion. After some discussion and deliberation during the procedural task, faculty suggested that “this is what stent you use and this is why” (P4).

#### Projection of personal characteristics

Communicating on a remote platform is unnatural and requires more work. When operating autonomously on a simulated patient with the consultant providing remote performance feedback, it is critical to confirm that the instruction given to you is the instruction you heard. One strategy to mitigate this is the use of closed-loop communication; one participant mentioned that they were “double checking that this was what they wanted before actually going ahead with it” (P2). The absence of non-verbal cues can impede communication and the subsequent flow of the operation, as remarked by one participant here, “I wasn't sure if they could see that I checked the wire placement” (P5).

Some participants emphasised the significance of recognising nonverbal cues and signs from faculty during the in-person course in order to establish a social connection with the trainer. Physical presence can also indicate if the “trainer is relaxed or if they feel on edge because you are not doing something quite right” (P2). One strategy to alleviate this is “if you could see their face” (P8), by employing a system that allows a two-way visual. However, other participants felt that forming a cohesive learning community “depends on your relationship with the specific trainer” (P10), as opposed to the setup of either platform for obtaining feedback.

#### Critical reflection and concept exchange

An individualist approach to learning was evident, this time from the perspective of task understanding, with one participant stating “it was maybe checking that what I was doing was right before proceeding with it, which is probably a better way to learn” (P2). Participants also expressed that the remote session was “less daunting” (P1), as it did not incur the physical presence of faculty, thus allowing a more uninhibited approach to knowledge assimilation.

Participants expressed varying perspectives on the visual and vocalisation conditions that trainers should convey prior to task commencement. Trainers who outlined engagement guidelines early in the session or from the start appeared to reduce ambiguity in participants and contribute to an overall enhanced learning experience. One participant highlighted concerns that faculty may miss opportunities to give feedback on technical steps because “they have missed a whole lot of errors that you are doing wrong” (P5), as they were unsure exactly what the faculty could see from the outset.

Technique illustration is a theme that was identified in our research inductively. However, it remains omitted from the modified CoI framework, as it requires the physical presence of the trainer throughout the learning experience to demonstrate skills such as “spinning the catheter” (P6), thus forming part of in person training only. It also cites physical demonstration as a potential obstacle to remote acquisition of certain skills, since it may make knowledge transfer via a remote medium challenging.

Participants advocated for extra training opportunities, either platform of feedback was reasonable, that “the biggest thing is the hands on, using the simulator” (P10). Notwithstanding the fact that there are unfamiliar feelings associated with learning via a remote platform, that residents and faculty must accustom to. Learning remotely “isn’t difficult, it’s just a change” (P11) from the normal learning environment in the OR.

### Questionnaire results

Median (IQR) responses for the 15 items selected from the CoI survey instrument ranged between 4 and 4.5 (agree). Item 8 (I felt comfortable interacting with the trainer) in social presence had the highest overall median score. As all groups provided survey responses just for the remote feedback segment, there were no statistically significant differences between the three groups reported based on the order of instruction, in person versus remote.

### CoI framework

Our research reports three modifications to the CoI framework (Fig. 1) previously reported by Cheng et al. for EV simulation training. Surgical technique is fostered primarily by feedback and discourse throughout task performance rather than afterwards in the debrief phase; therefore, for EV simulation education training, we have modified these components of the framework to reflect the following: “instructional frameworks”, “continuous discourse” and “training experience”.

## Discussion

Studies carried out using carotid stenting and peripheral vascular angioplasty procedures demonstrate that simulator training is a valid, feasible, and acceptable training tool, and skills learned on simulators are transferable to the OR [27]. In order to increase opportunities for training, the delivery of existing programmes needs to be reconsidered. There is currently a paucity of research on the delivery training for EV techniques supported by remote instructional feedback, thus requiring further investigation. Our study used interviews and a modified CoI questionnaire to compare the experience of vascular surgery residents receiving both remote and in-person feedback for EV training.

Overall, the data elicited from our interviews and questionnaire suggests that remote feedback is an effective platform to support vascular surgeons training in EV technique; both participants and trainers acclimated to the remote course reasonably quickly. In the educator and cognitive elements of the CoI framework, responses to the questionnaire were similar and comparable across all groups. According to the survey, group 2 felt slightly less at ease with the social segregation of the trainer, and this was unsurprising given that their first attempt at completing the procedure was with the trainer providing feedback remotely, requiring them to quickly adapt to alien learning surroundings while carrying out the task at hand, though neither group reported significant statistical difference in terms of the effectiveness of either feedback platform. Providing and receiving remote feedback require a distinct skills set that both trainers and residents must become proficient in, if it is to become a viable platform for delivering EV SBE programmes. Educators should recognise the effects of hierarchy on comfort level of learners. Creating and maintaining psychological safety are key to a collaborative learning environment.

Some participants expressed apprehension during the remote course as they were unsure what exactly the trainers could see, or it took a short period of time following task commencement for it to become apparent to them what the trainers could see. According to our survey, most participants agreed that the measures taken by the trainers promoted a sense of community amongst both groups, and a detailed pre-brief that includes components unique to EV simulation-based learning would mitigate this. For example, in order to allow residents to effectively accomplish EV tasks in a remote setting, trainers must be able to detect intricate hand movements and observe task progression through essential clinical and anatomical characteristics. It is important for learners to be aware of the specific camera angles that trainers have on the simulation arena in which these EV procedures are observed, as this will serve to reduce uncertainty and strengthen the sense of community between both groups. Other healthcare fields that have published specifics of their remote simulation-based learnings in the literature may not place a premium on these features [28]. Fields of view is an important consideration for the orientation to the learning environment and should be outlined in the pre-brief as part of the rules of engagement [14] to alleviate the resident-trainer disconnect.

The concept of conversational turn taking arose in this study, principally with respect to challenges faced around discontinuous progression of the task. According to recent research, the absence of regular positive social interactions on remote platforms leads to a decline in conversational turn taking [29]. It is important to recognise this as a characteristic of a remote teaching environment and make attempts to ameliorate it while preserving a psychological safety [30, 31]. The use of organised turn taking is one strategy for encouraging continuous discourse and avoiding awkward pauses, with cues directing this approach [32]. Any conditions or strategies for more effective conversational turn taking should be made explicit as rules of engagement from the outset, in order to avoid any confusion.

We used an AV system that had a one-way visual capacity, which enabled the trainer to observe the resident but not the other way around. Some participants emphasised that effective communication was slightly hindered by the trainer’s physical absence from social interactions during the remote course. However, other participants preferred the trainer’s physical absence in the remote course, with the times of contemplation allowing them to digest cognitive leaps calmly and relish the challenges associated with learner independence [33]. There are cost-effective technologies available online, which provide trainers and residents with a two-way visual learning experience that aids in the recognition of nonverbal signs and, as a result, encourages more cohesive learning groups.

## Conclusions

Participants in general emphasised the significance of gaining hands-on simulator experience and are open to expert feedback on either platform. Overall, the survey highlights and data elicited from interviews suggest that the remote platform is quite an effective method in obtaining the necessary instruction to complete EV procedural tasks and would be more feasible in attracting a local and a wider pool of experts to teach. This study provides a solid foundation of knowledge on what the requirements are to safely hone EV psychomotor skills in a simulated environment with remote instructional feedback. Future research should build on this work by eliciting information about the implementation of remote EV SBE programmes.

### Strengths and limitations

One of the study’s strengths was that it included feedback from four different vascular surgeons, providing a variety of techniques to providing feedback. Our research reports a number of limitations. Firstly, we used a relatively small sample size, owing to the small population of vascular surgery residents in the Republic of Ireland. More participants perhaps may have resulted in a more in-depth conversation and understanding of the topic. Secondly, participants performed the same procedural task in both settings, with the goal of revising and cementing learnings from the first session. Perhaps a different task in each session would have resulted in a better nuanced understanding. Nonetheless, participants were able to compare like with like by performing the identical task in both sittings. Lastly, most virtual platforms have two-way visual technology enabled; therefore, we acknowledge that perhaps we may have yielded additional information on remote feedback with this technology in place.

### Supplementary Information

Supplementary Material 1: Appendix A. Modified task specific procedural checklist.

## Data Availability

The datasets generated and analysed during the current study are not publicly available due to stipulations set out in the participant information leaflet but are available from the corresponding author on reasonable request.

## Abbreviations

SBE: Simulation-based education
EV: Endovascular
VR: Virtual reality
CoI: Community of inquiry
RCSI: Royal College of Surgeons Ireland
AV: Audio-visual
SIM: Simulation
NCHD: Non-consultant hospital doctors
RRAS: Right renal artery stenosis
OR: Operating room
Pre-op: Pre-operative
