# Supplementary material for: Remote feedback in endovascular simulation training: a mixed-methods study
Source: Adv Simul (Lond). 2024 Jun 11;9:24. doi: 10.1186/s41077-024-00297-0 (PMC11165733; doi:10.1186/s41077-024-00297-0)
Supplement: Supplementary file 1 — Supplementary Material 1: Appendix A. Modified task specific procedural checklist. [file 41077_2024_297_MOESM1_ESM.docx]

**Appendix A:** Modified task specific procedural checklist

# Renal artery stenosis intervention

Participant id:

Date:

| **Performance criteria:** | **Suboptimal** | **Adequate** | **Good** |
| --- | --- | --- | --- |
| Indications for renal artery stenting |  |  |  |
| Suitability of guidewires used throughout |  |  |  |
| Suitability of catheters used throughout |  |  |  |
| **LESION** |  |  |  |
| Placement accuracy distal (centre lesion – centre balloon) |  |  |  |
| Diameter of lesion stenosed (before dilation) |  |  |  |
| Diameter of balloon (inflated) |  |  |  |
| % of lesion covered with balloon |  |  |  |
| Stent – vessel ratio |  |  |  |
| **HANDLING EVENTS** |  |  |  |
| Diagnostic catheter scraping against vessel wall |  |  |  |
| Diagnostic catheter moving without support of wire |  |  |  |
| Diagnostic catheter too deep into ostium |  |  |  |
| Guide wire in small branch |  |  |  |
| Catheter scraping against vessel wall |  |  |  |
| Catheter moving without support of wire |  |  |  |
| Catheter entered suboptimal vessel |  |  |  |
| Guide wire entered suboptimal vessel |  |  |  |
| **TIMES** |  |  |  |
| Use of DSA |  |  |  |
| Use of Cine |  |  |  |
| Use of fluoroscopy |  |  |  |
| Total amount of contrast used |  |  |  |
| Total time to perform the task |  |  |  |

| *Overall competence* | **Not competent / fail** | **Borderline** | **Competent / pass** |
| --- | --- | --- | --- |
| **Global score** |  |  |  |
